# Using Portuguese *BRCA* pathogenic variation as a model to study the impact of human admixture on human health

**DOI:** 10.1186/s12864-024-10311-4

**Published:** 2024-04-27

**Authors:** Stephanie Andaluz, Bojin Zhao, Siddharth Sinha, Philip Naderev Panuringan Lagniton, Diogo Alpuim Costa, Xiaofan Ding, Miguel Brito, San Ming Wang

**Affiliations:** 1 Ministry of Education Frontiers Science Center for Precision Oncology, Cancer Center and Institute of Translational Medicine, Faculty of Health Sciences, University of Macau, Macao SRA, China; 2 Medical Oncology Department, Hospital de Cascais, Cascais; Haematology and Oncology Department, CUF Oncologia, Lisbon; NOVA Medical School, Faculdade de Ciências Médicas, Universidade NOVA de Lisboa, Lisbon, Portugal; 3 Escola Superior de Tecnologia da Saúde de Lisboa, Instituto Politécnico de Lisboa, Lisbon, Portugal

**Keywords:** Admixture, *BRCA1*, *BRCA2*, Pathogenic variants, Cancer risk, Portuguese, Brazilian

## Abstract

**Background:**

Admixture occurs between different ethnic human populations. The global colonization in recent centuries by Europeans led to the most significant admixture in human history. While admixture may enhance genetic diversity for better fitness, it may also impact on human health by transmitting genetic variants for disease susceptibility in the admixture population. The admixture by Portuguese global exploration initiated in the 15^th^ century has reached over 20 million of Portuguese-heritage population worldwide. It provides a valuable model to study the impact of admixture on human health. *BRCA1* and *BRCA2* (*BRCA*) are two of the important tumor suppressor genes. The pathogenic variation (PV) in *BRCA* is well determined to cause high risk of hereditary breast and ovarian cancer. Tracing the distribution of Portuguese *BRCA* PV in Portuguese-heritage population will help to understand the impact of admixture on cancer susceptibility in modern humans. In this study, we analyzed the distribution of the Portuguese-originated *BRCA* variation in Brazilian population, which has high degree Portuguese-heritage.

**Methods:**

By comprehensive data mining, standardization and annotation, we generated a Portuguese-derived *BRCA* variation dataset and a Brazilian-derived *BRCA* variation dataset. We compared the two *BRCA* variation datasets to identify the *BRCA* variants shared between the two populations.

**Results:**

The Portuguese-derived *BRCA* variation dataset consists of 220 *BRCA* variants including 78 PVs from 11,482 Portuguese cancer patients, 93 (42.2%) in *BRCA1* and 127 (57.7%) in *BRCA2*. Of the 556 Portuguese *BRCA* PV carriers carrying the 78 PVs, 331 (59.5%) carried the three Portuguese-*BRCA* founder PVs of *BRCA1* c.2037delinsCC, *BRCA1* c.3331_3334del and *BRCA2* c.156_157insAlu. The Brazilian-derived *BRCA* variation dataset consists of 255 *BRCA* PVs from 7,711 cancer patients, 136 (53.3%) in *BRCA1* and 119 (46.6%) in *BRCA2*. We developed an open database named dbBRCA-Portuguese (https://genemutation.fhs.um.edu.mo/dbbrca-portuguese/) and an open database named dbBRCA-Brazilian (https://genemutation.fhs.um.edu.mo/dbbrca-brazilian) to host the *BRCA* variation data from Portuguese and Brazilian populations. We compared the *BRCA* PV datasets between Portuguese and Brazilian populations, and identified 29 Portuguese-specific *BRCA* PVs shared between Portuguese and Brazilian populations, 14 in *BRCA1* including the Portuguese founder *BRCA1* c.3331_3334del and *BRCA1* c.2037delinsCC, and 15 in *BRCA2* including the Portuguese founder *BRCA2* c.156_157insAlu. Searching the 78 Portuguese *BRCA* PVs in over 5,000 ancient human genomes identified evolution origin for only 8 PVs in Europeans dated between 37,470 and 3,818 years before present, confirming the Portuguese-specificity of Portuguese *BRCA* PVs; comparing the 78 Portuguese *BRCA* PVs Portuguese, 255 Brazilian *BRCA* PVs, and 134 African BRCA PVs showed little overlapping, ruling out the possibility that the *BRCA* PVs shared between Portuguese and Brazilian may also be contributed by African.

**Conclusion:**

Our study provides evidence that the admixture in recent human history contributed to cancer susceptibility in modern humans.

**Supplementary Information:**

The online version contains supplementary material available at 10.1186/s12864-024-10311-4.

## Background

Modern humans originated from Africa 100,000 - 60,000 years ago and migrated to different global destinations [1]. Genetic variation in different environments has resulted in differential variation signatures between different ethnic populations. During the process of human evolutionary history, admixture between geographically isolated populations occurred by means of migration, occupation, displacement, and war etc. [2]. However, the global colonization by Europeans in recent hundreds of years has resulted in the most widespread admixture in human history [3]. Biomedically, admixture could not only enhance genetic diversity, but may also transfer disease susceptibility between populations [4, 5]. The impact of admixture on human health gains increased attention [6, 7]. For example, modern humans inherited multiple loci in chromosome 3 from the extinct Neanderthals, which can regulate the expression of *CCR1* and *CCR5,* the critical chemokine receptor genes involving the severe immune response to COVID-19 infection [8, 9]; the pathogens carried by Europeans to the New World caused catastrophic consequence on Native Americans [10]. However, most of the previous studies focused on the susceptibility for infectious diseases. Little is known for the impact of admixture on susceptibility for chronic diseases, such as cancer, in modern humans.

**Fig. 1:**
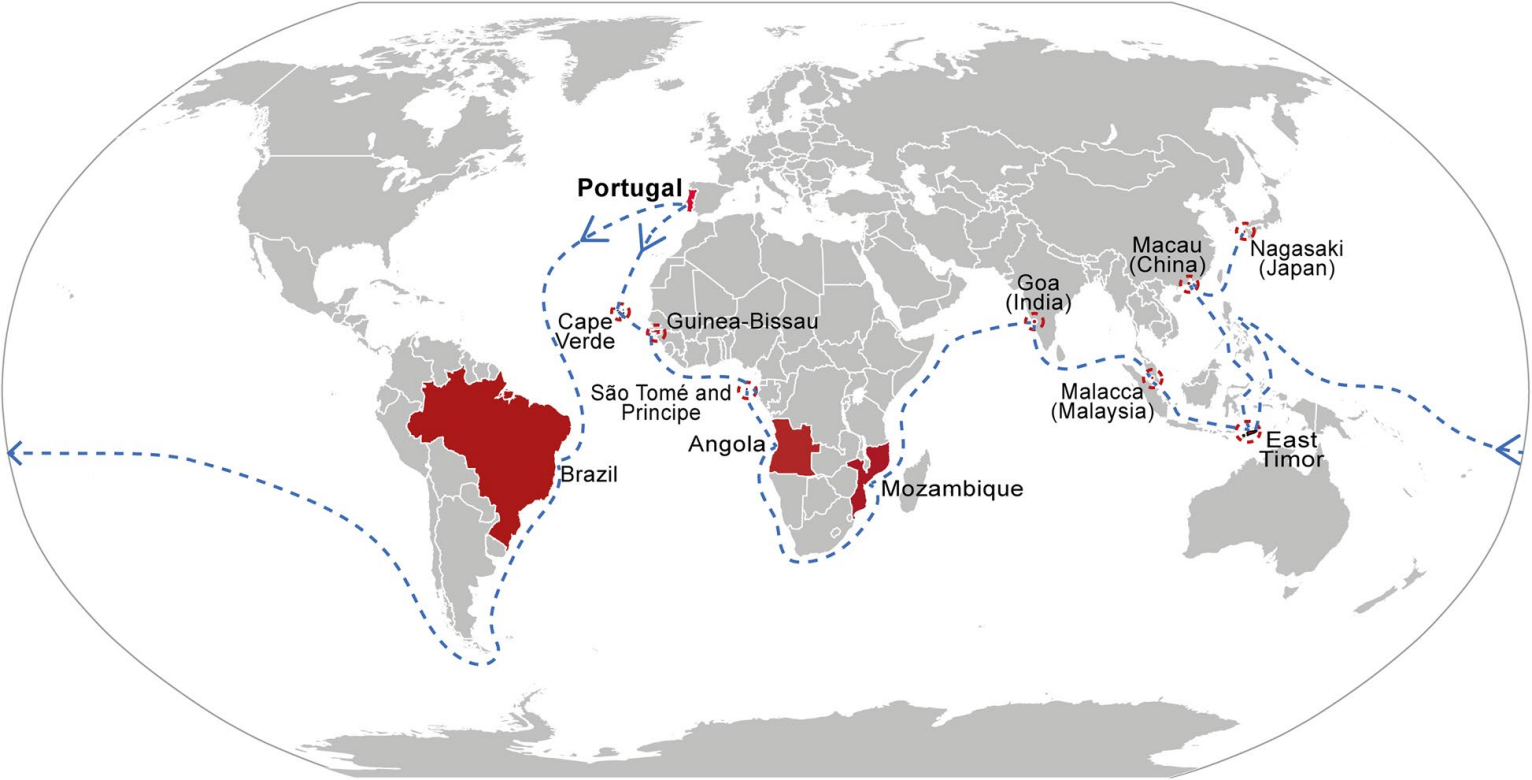
Portuguese global exploration since the 15^th^ century. It outlines the East path towards Asia and the West path towards South America and the Pacific. Red: Portuguese colonized countries and regions

The Portuguese global exploration provides a typical example of human admixture. Initiated in the 15^th^ century, the Portuguese established its colonies across Africa, Asia and America, including countries of Angola, Mozambique, India, Sri Lanka, Malaya, China, Japan, East Timor and Brazil etc. [11] (Fig. 1). In many of the places colonized by Portuguese, admixture formed between Portuguese and locals. Up to now, there are over 20-million Portuguese-heritage population worldwide in comparing to 10.3-million native Portuguese in Portugal [12] (Table S1). For example, around five-million Brazilian individuals contain Portugal heritage constituting 2.3% of Brazil population, over 8,000 Macau individuals named Macanese contain Portugal heritage constituting 1.5% of Macau population [13]. The population with Portuguese heritage provides a valuable model to study the impact of admixture on human health.

Genomic DNA in the genome is constantly damaged by external and internal factors. The damaged DNA must be repaired in order to prevent genome instability, which otherwise can cause high susceptibility to diseases, in particular cancers. This function is achieved by the DNA damage repair (DDR) system consisting of multiple pathways and hundreds of different genes. The pathways include homologous recombination (HR) and nonhomologous end joining (NHEJ) pathways repairing double strand DNA breaks; base excision repair (BER) pathway repairing small, non-helix–distorting base lesions; direct reversal (DR) repair pathway repairing the DNA damaged by ubiquitous alkylating agents; Fanconi anemia (FA) pathway repairing strand cross-link errors; mismatch repair (MMR) pathway repairing mismatch errors; and nucleotide excision repair (NER) pathway repairing helix-distorting DNA lesions. The joint activities of a group of genes in each DDR pathway repair the given type of DNA damage [14]. For example, in homology recombination pathway, BRCA1 interacts with PALB2 and BRCA2/RAD51 in the double strand break sites to initiate the repair process, and BRCA2 interacts with RAD51 to single-stranded DNA to promote DNA strand exchange and homologous pairing to repair the damage DNA [15, 16].

However, DDR genes themselves are prone to genetic variation. The variation with pathogenic effects can damage the function of the affected genes, leading to disease susceptibility. For example, women carrying pathogenic variants (PVs) in *BRCA1* have a 55%–72% lifetime risk of developing breast cancer, 39%–44% lifetime risk of developing ovarian cancer; women carrying pathogenic variants in *BRCA2* have a 45%–69% risk of developing breast cancer and 11%–17% lifetime risk of developing ovarian cancer [17–19]. PVs in *BRCA1* and *BRCA2* also increase the risk of developing prostate cancer, colorectal cancer and pancreatic cancer [20]. Identification of *BRCA* PV carriers without cancer can prevent cancer development by taking preventive measures through early cancer surveillance, chemoprevention and preventive surgery [21–25], and identification of *BRCA* PV carriers with cancer can benefit the use of targeted therapy, such as PARP inhibitors, to treat the cancer [26]. In our current study, we targeted *BRCA1* and *BRCA2* (*BRCA*) based on the following considerations: 1). High clinical significance. It is well determined that *BRCA* PV is highly linked to cancer risk, and is widely used in clinical practice as the genetic marker for cancer diagnosis, prevention, prognosis, and treatment [27–31]. Therefore, information from *BRCA* study could be highly significance for clinical applications; 2). High prevalence. among all DDR genes, *BRCA* PV has the highest prevalence that *BRCA1* PVs reaches 0.079% and *BRCA2* PVs 0.124% in worldwide human populations, whereas the prevalence of the PVs other DDR genes was much lower, e.g., 0.034% in *CHEK2*, 0.015% in *TP53*, 0.010% in *RAD51D*, and 0.002% in *POLK* [32]. The higher prevalence of *BRCA* PVs implies higher relevance of the results to human health; 3). Availability of rich variation data. *BRCA* was among the first determined cancer risk genes [33]. Extensive efforts made in the past decades have identiifed over 70,000 genetic variants in human *BRCA* [34] (https://brcaexchange.org/factsheet). Importantly, *BRCA* variation has also been extensively characterized in both Portuguese population and Portuguese heritage-rich Brazilian population.

In current study, we used the *BRCA* PVs from Portuguese and Brazilian populations as a model to study the impact of admixture on cancer susceptibility. By comprehensive collection, standardization and classification of the *BRCA* variation data originated from Portuguese and Brazilian populations, we generated the centralized *BRCA* variation data in both Portuguese and Brazilian populations. By comparing the *BRCA* PV data between Portuguese and Brazilian populations, we observed the 0 presence of Portuguese-originated *BRCA* PVs variation in Brazilian population. Our study provides evidence showing the impact of admixture on human cancer susceptibility.

## Results

### *BRCA* variation in Portuguese population

**Fig. 2:**
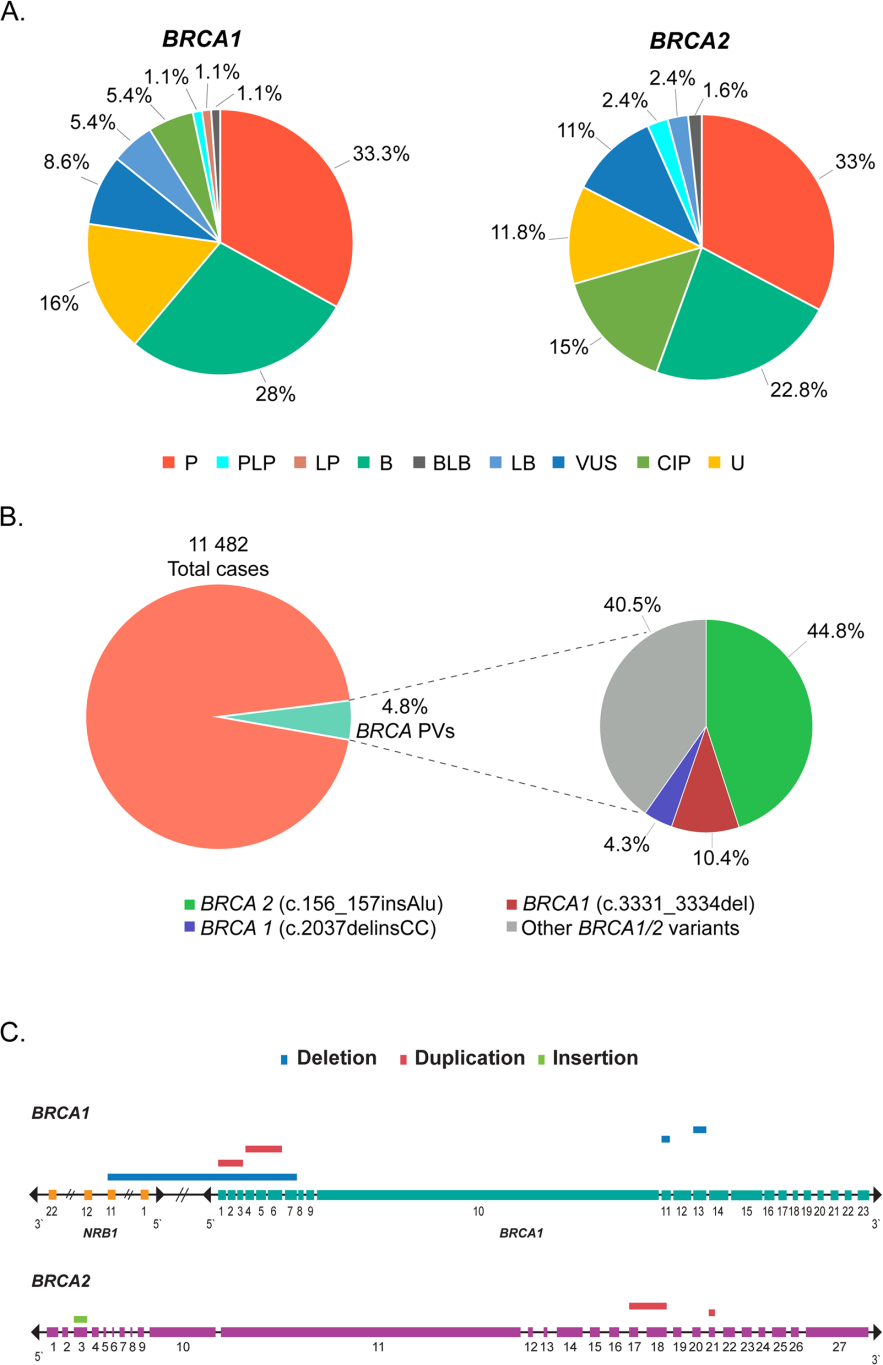
Features of Portuguese *BRCA* variation. **A** Clinical classification of *BRCA* variants. P: Pathogenic; PLP: Pathogenic or Likely Pathogenic; LP: Likely Pathogenic; B: Benign; LB: Likely Benign; BLB: Benign or Likely Benign; VUS: Variants of Uncertain Significance; CIP: Conflicted Interpretation of Pathogenicity; U: Unknown. **B** *BRCA* PVs identified in Portuguese cancer cohort. The left pie showed 4.8% of 11,482 Portuguese cancer patients were the *BRCA* PV carriers; the right pie showed that 59.5% of the PV carriers carried the three Portuguese founder pathogenic variants, and 40.5% carried non-founder pathogenic variants. **C** Large structural variants in Portuguese *BRCA* variation

We first generated a comprehensive collection of Portuguese**-**originated *BRCA* variation data. We identified a total of 23 publications between 2000 and 2022 reporting the *BRCA* variation data in 11,482 Portuguese cancer patients, mostly breast cancer, ovarian cancer, prostatic cancer, and HBOC (Hereditary Breast and Ovarian Cancer Syndrome), largely reported by the Laboratory of Dr. Manual Teixeira in Portuguese Oncology Institute of Porto, Portugal ([35–57], Table S1). We extracted the *BRCA* variation data from the publications. Upon standardization, annotation and integration, we identified a total of 220 non-redundant *BRCA* variants, including 93 (42.3%) in *BRCA1* and 127 (57.7%) in *BRCA2*. By referring to ClinVar database, the 220 *BRCA* variants were classified into different clinical classes, including 78 (35.5%) Pathogenic or Likely Pathogenic variants (PV), 66 (30.0%) Benign and Likely Benign variants (BV), 55 (25%) Variant of Unknown Significance (VUS), 24 (10.9%) conflict of interpretation, and 30 (13.6%) unclassified variants (Table 1A, Fig. 2A, B, Table S2). Single base variation accounted for the majority of variants (65.4%) (Table S5). In the 11,482 cancer patients tested in the original studies, 556 (4.8%) cases were identified as the carriers for the 78 *BRCA* PVs (Fig. 2B). We also identified 8 large structural variation, 5 in *BRCA1* of one deletion covering *NRB1* exon 1 to exon 11 and *BRCA1* exon 1 to exon 7, one duplication covering exon 1 to exon 3, one duplication covering exon 4 to exon 6, one deletion affecting exon 11 and one deletion affecting exon 13; and three in *BRCA2* of one insertion in exon 3, one duplication from exon 17 and exon 18, and one duplication in exon 21 (Fig. 2C, Table S3, Table S4).

**Table 1:** *BRCA* variants identified in Portuguese

A. Clinical classification of the *BRCA* variants						
	P/LP	B/LB	VUS	Conflicts	Unknown	Total
*BRCA1*	33	32	8	5	15	93
*BRCA2*	45	34	14	19	15	127
Total	78	66	22	24	30	220
B. Portuguese-specific *BRCA* PVs						
cDNA	Protein	Variant type	ClinVar	Tested cases	Carrier	Carrier rate (%)
***BRCA1***						
c.3323_3326del	p.Ile1108Lysfs^a^8	Deletion	P	112	2	1.79
**c.2037delinsCC**^**a**^	**p.Lys679Asnfs**^**a**^**4**	**Indel**	**P**	**1,595**	**24**	**1.50**
c.2309C>A	p.Ser770^a^	SNV	P	94	1	1.06
c.3477_3480del	p.Ile1159Metfs^a^50	Deletion	P	94	1	1.06
c.4165_4166del	p.Ser1389^a^	Microsatellite	P	94	1	1.06
c.211A>G	p.Arg71Gly	SNV	P	1,598	12	0.75
c.116G>T	p.Cys39Phe	SNV	P/LP	135	1	0.74
c.1016dup	p.Val340Glyfs^a^6	Duplication	P	135	1	0.74
c.1058G>A	p.Trp353^a^	SNV	P	135	1	0.74
c.4485-2A>C	p.?	SNV	LP	135	1	0.74
c.3817C>T	p.Gln1273^a^	SNV	P	1,360	9	0.66
**c.3331_3334del**^**a**^	**p.Gln1111Asnfs**^**a**^**5**	**Deletion**	**P**	**10,337**	**58**	**0.56**
c.470_471del	p.Ser157^a^	Microsatellite	P	1,364	7	0.51
c.536del	p.Tyr179fs	Deletion	P	210	1	0.48
c.2086dup	p.Thr696Asnfs^a^16	Duplication	P	1,170	3	0.26
c.4484G>T	p.Arg1495Met	SNV	P	1,195	3	0.25
c.66dup	p.Glu23Argfs^a^18	Duplication	P	1,270	3	0.24
c.2418del	p.Ala807Hisfs^a^8	Deletion	P	1,264	3	0.24
c.1969C>T	p.Gln657^a^	SNV	P	1,270	3	0.24
c.3481_3491del	p.Glu1161Phefs^a^3	Deletion	P	1,270	3	0.24
c.3627dup	p.Glu1210Argfs^a^9	Duplication	P	1,270	3	0.24
c.4136_4137del	p.Ser1379^a^	Microsatellite	P	1,270	3	0.24
c.2490_2497dup	p.Leu833Cysfs^a^16	Duplication	P	1,170	2	0.17
c.2906del	p.Asn969Ilefs^a^31	Deletion	P	1,170	2	0.17
c.3257T>A	p.Leu1086^a^	SNV	P	1,170	2	0.17
c.4891del	p.Ser1631Valfs^a^2	Deletion	P	1,170	2	0.17
***BRCA2***						
**c.156_157insAlu**^**a**^	**p.Lys53delinsAlaGlyArgGlyArgSerArgLeuTer**	**Insertion**	**P**	**11,574**	**249**	**2.15**
c.2T>G	p.Met1Arg	SNV	P	215	4	1.86
c.5934dup	p.Ser1979Ter	Duplication	P	94	1	1.06
c.6656C>G	p.Ser2219Ter	SNV	P	94	1	1.06
c.7738C>T	p.Gln2580Ter	SNV	P	94	1	1.06
c.9004G>A	p.Glu3002Lys	SNV	P/LP	94	1	1.06
c.7208_7211del	p.Thr2403fs	Microsatellite	P	210	2	0.95
c.7975A>G	p.Arg2659Gly	SNV	P	325	3	0.92
c.8642_8729dup	p.Ala2911LysfsTer25	Duplication	P	112	1	0.89
c.8866_8988-2133dup	p.?	Insertion	P	112	1	0.89
c.5436del	p.Glu1812AspfsTer3	Deletion	P	135	1	0.74
c.8488-1G>A	p.?	SNV	P	1,426	7	0.49
c.1310_1311del	p.Lys437ArgfsTer14	Deletion	P	210	1	0.48
c.1423G>T	p.Glu475Ter	SNV	P	210	1	0.48
c.6468_6469del	p.Gln2157fs	Deletion	P	210	1	0.48
c.9098_9099insA	p.Gln3034fs	Insertion	P	210	1	0.48
c.682-2A>C	p.?	SNV	P/LP	1,195	5	0.42
c.5645C>A	p.Ser1882Ter	SNV	P	1,170	4	0.34
c.8954-5A>G	p.?	SNV	P/LP	1,195	3	0.25
c.5355dup	p.Ser1786Ter	Duplication	P	1,270	3	0.24
c.8695C>T	p.Gln2899Ter	SNV	P	1,270	3	0.24
c.9382C>T	p.Arg3128Ter	SNV	P	4,857	11	0.23
c.4808del	p.Asn1603ThrfsTer14	Deletion	P	1,150	2	0.17
c.956del	p.Asn319IlefsTer5	Deletion	P	1,170	2	0.17
c.1368_1369dup	p.Lys457ArgfsTer4	Microsatellite	P	1,170	2	0.17
c.4380_4381del	p.Ser1461LeufsTer4	Deletion	P	1,170	2	0.17
c.5653dup	p.Cys1885LeufsTer15	Duplication	P	1,170	2	0.17
c.5722_5723del	p.Leu1908ArgfsTer2	Microsatellite	P	1,170	2	0.17
c.7007G>A	p.Arg2336His	SNV	P	1,170	2	0.17
c.7631del	p.Gly2544AlafsTer7	Deletion	P	1,170	2	0.17

^a^Portuguese *BRCA* founder variants

### Portuguese *BRCA* founder variants

Founder variant is defined as “a pathogenic variant observed at high frequency in a specific population due to the presence of the variant in a single ancestor or small number of ancestors [58]. Three *BRCA* PVs are well determined as Portuguese *BRCA* founder variants, including *BRCA1* c.2037delinsCC, *BRCA1* c.3331_3334del, and *BRCA2* c.156_157insAlu [59]. Of the 556 *BRCA* PV carriers identified in the 11,482 cancer patients, 331(59.5%) were the carriers for the three founder variants, including 24 (4.3%) carriers of *BRCA1* c.2037delinsCC, 58 (10.4%) carriers of *BRCA1* c.3331_3334del, and 249 (44.8%) carriers of *BRCA2* c.156_157insAlu (Fig. 2B).

### Portuguese-specific *BRCA* PVs

We searched the Portuguese-originated *BRCA* PVs in GnomAD database v.3.1.2, which contained genetic variation data from 76,156 individual genomes of African, African American, Amish, Ashkenazi Jewish, East Asian, Middle Eastern, South Asian, Latino, Admixed American, Finnish European, and non-Finnish European. We observed that 56 (71.8%) of the 78 Portuguese *BRCA* PVs were not present in non-Portuguese populations (Table 1B), the remaining 23 PVs (29.1%), they were mainly shared with the non-Finnish European populations (Table S5).

### *BRCA* variation in Brazilian population

We identified 24 publications reporting *BRCA* variation in 7,711 Brazilian individuals mostly breast cancer, ovarian cancer and HBOC patients (Table S6A). We extracted the *BRCA* variants from the reports, and performed standardization, annotation, and classification. We identified a total of 255 *BRCA* PVs, 136 (53.3%) in *BRCA1* with 743 carriers and 119 (46.6%) in *BRCA2* with 365 carriers (Table S6B, Table S6C). The carrier rate of *BRCA* PV was about 3.3% in the tested 7,711 Brazilian cancer patients.

### *BRCA* variation databases

We developed two open-access databases, one is the dbBRCA-Portuguese to host the Portuguese-originated *BRCA* variation data (https://genemutation.fhs.um.edu.mo/dbbrca-portuguese/) and the other is the dbBRCA-Brazilian to host the Brazilian-originated *BRCA* variation data (https://genemutation.fhs.um.edu.mo/dbbrca-brazilian/). The databases provide the information on variants, annotation, clinical classification, cancer type, carrier frequency, and original references etc., users can perform online search or download the entire datasets for their own analysis.

### Presence of Portuguese *BRCA* PVs in Brazilian population

**Fig. 3:**
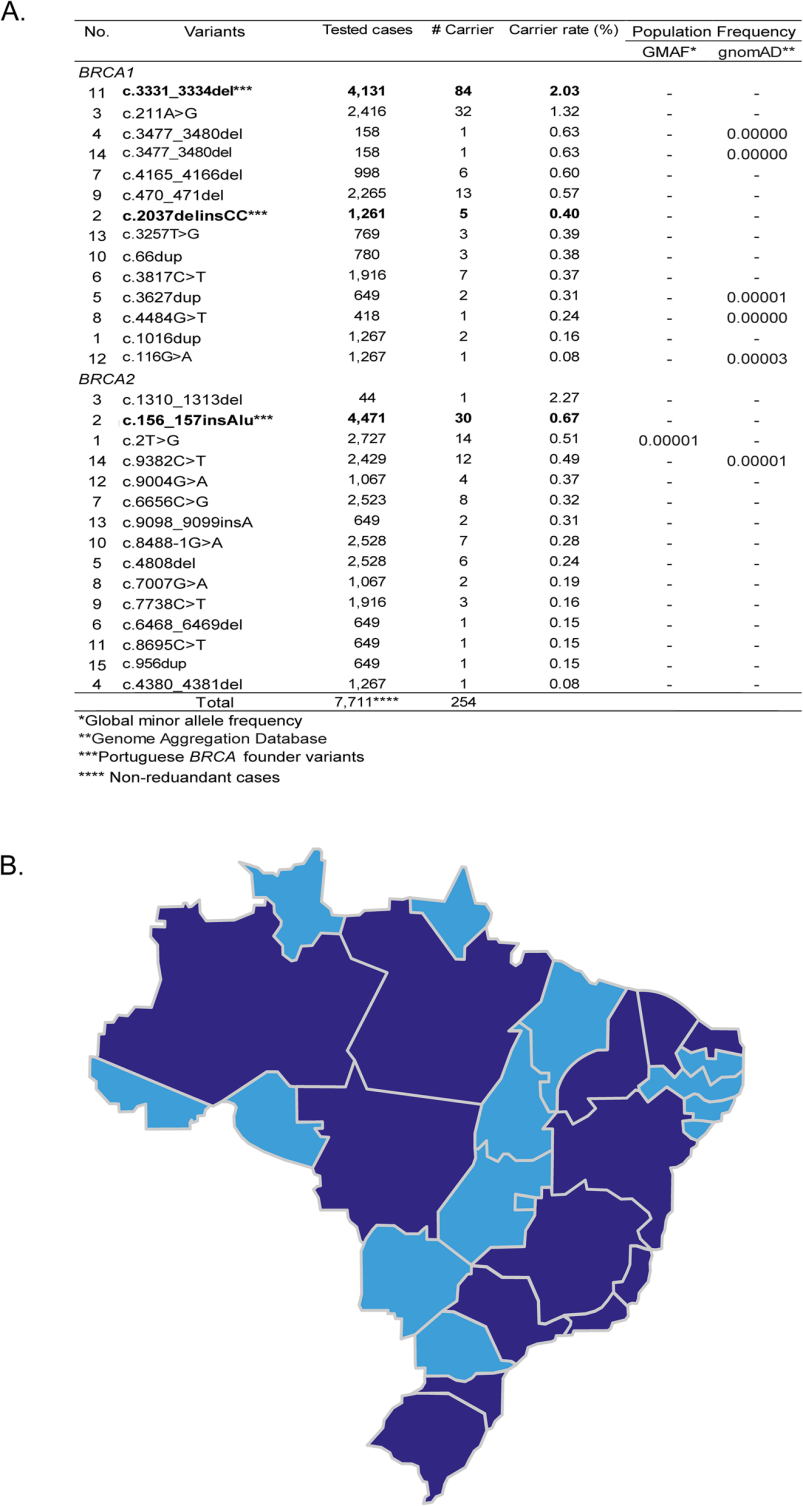
Portuguese-specific *BRCA* PVs in Brazilian population. **A** 29 of the 56 Portuguese-specific *BRCA* PVs identified in Brazilian population; **B** Resident location of the Brazilian carriers of Portuguese *BRCA* PVs in the states of Brazil (Darker blue)

Brazil has a population size of over 215 million, of which 5 million (2.3%) has Portuguese-heritage background (https://data.worldbank.org/indicator/SP.POP.TOTL?locations=BR). We tested if the Portuguese *BRCA* PVs could be present in Brazilian population. To ensure high reliability of the results, we used only the 56 Portuguese-specific *BRCA* PVs for the comparison. We observed that 29 (51.8%) of the 56 Portuguese-specific *BRCA* PVs were present in Brazilian population with 254 carriers, of which 119 (46.7%) carried the three Portuguese founder variants, including 14 in *BRCA1* including the Portuguese founder *BRCA1* c.3331_3334del [84 carriers in 4,131 (2.03%) tested individuals], *BRCA1* c.2037delinsCC [5 carriers in 1,261 (0.4%) tested individuals], and 15 in *BRCA2* including the Portuguese founder *BRCA2* c.156_157insAlu [30 in 4,471 (0.67%) tested individuals]. There were 254 carriers in the 7,711 tested Brazilian cancer patients, reaching to the carrier frequency of 3.2% (Fig. 3A, Table S6B, Table S6C). Searching the shared PVs in Global Minor Allele Frequency (GMAF) and Genome Aggregation Databases (gnomAD) via ClinVar showed that these shared variants had either no or low population frequency information, indicating that they were all rare variants (Fig. 3A). None of the eight large BRCA structural variations in Portuguese were identified in Brazilian. The Brazilian carriers for the 29 shared *BRCA* PVs were residents in 13 of the 26 States and 1 Federal District of Brazil (Fig. 3B).

**Fig. 4:**
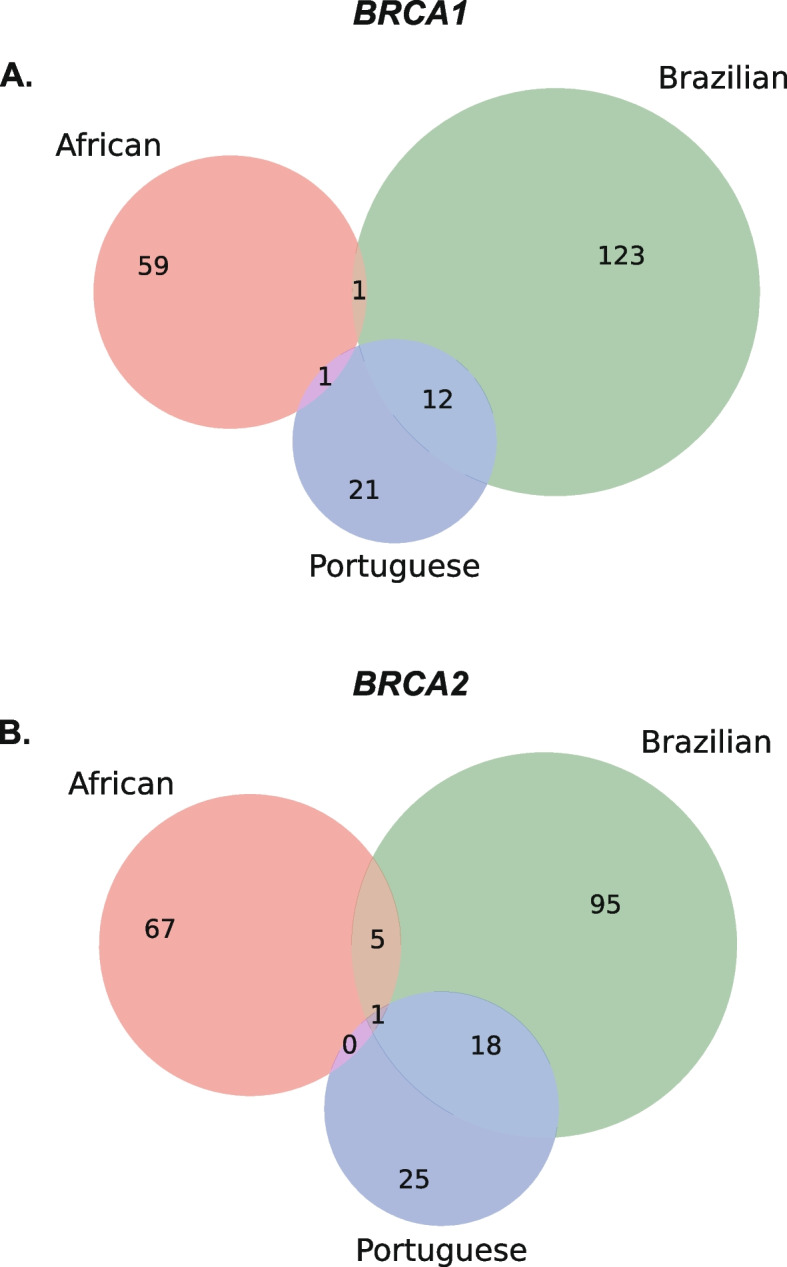
Comparison of the *BRCA* PVs between African, Brazilian, and Portuguese. The PVs included 134 African *BRCA* PVs, 255 *BRCA* PVs in Brazilian, and 78 *BRCA* PVs in Portuguese. Only 1 of the 136 Brazilian *BRCA1* PVs and 5 of the 119 Brazilian *BRCA2* PVs were present in African, and 1 of the 119 Brazilian *BRCA2* PVs was present in all three populations. The shared PVs were not Portuguese founder PVs, and not included in the 56 Portuguese-specific *BRCA* PVs

Our analysis of GenomAD database showed that the Portuguese*-*derived *BRCA* PVs were highly Portuguese-Specific, and widely present in Brazilian population. However, native American, African and European etc. all contribute to the genetic background for today’s Brazilian [60]. Besides the *BRCA* PVs in Brazilian originated from Portuguese (European), possibility exists that certain *BRCA* PVs might also be inherited from Native American or African. If this could be the case, assigning Portuguese as the sole source of *BRCA* PVs would not be appropriate. *BRCA* variation has not been systematically tested in Native American, which restricts the analysis for the relationship of BRCA PVs between native American and Brazilian. However, *BRCA* variation data from African are available. This allows us to test the relationship between Portuguese, Brazilian and African. We compared the *BRCA* PVs between African, Brazilian, and Portuguese. The PVs included the 134 African *BRCA* PVs (61 in *BRCA1* and 73 in *BRCA2*) identified by CIMBA (the Consortium of Investigators of Modifiers of BRCA1/2) study [61], the 255 *BRCA* PVs in Brazilian, and the 78 *BRCA* PVs in Portuguese. The results showed that only 1 of the 136 (0.7%) Brazilian *BRCA1* PVs (c.2389_2390del), 5 of the 119 (4.2%) Brazilian *BRCA2* PVs (c.93G>A, 5197_5198del, c.5616_5620del, c.7900del, c.8009C>T) were present in African, and only 1 of the 119 (0.8%) Brazilian *BRCA2* PVs (c.2808_2811del) was present in all three populations (Fig. 4). None of the 7 shared PVs were the Portuguese founder PVs nor within the 56 Portuguese-specific *BRCA* PVs defined after filtering in GenomAD database (Fig. 1B). The results ruled out the possibility that African contributed to the *BRCA* PVs shared between Portuguese and Brazilian.

We also searched the evolution origin for the 78 *BRCA* PVs in Portuguese. From the genomic data derived from over 5,000 ancient humans, we identified only 8 (10%) of the 78 *BRCA* PVs (3 in *BRCA1* and 5 in *BRCA2*) present in ancient Europeans dated between 37,470 to 38,18 years before present, of which only *BRCA2* c.9004G>A, c.8488-1G>A and c.7007G>A were among the 56 Portuguese-specific *BRCA* PVs (Table 2). Although the 8 Portuguese PVs highlighted their European origin, the fact that 70 of the 78 *BRCA* PVs were not present in the ancient humans further confirmed their Portuguese-specificity.

**Table 2:** Evolutionary origin of the *BRCA* PVs in Portuguese

cDNA	Protein	Type	Location	Time (BP)^a^
*BRCA1*				
c.181T>G	p.(Cys61Gly)	nonsynonymous SNV	Russia	37470
c.1058G>A	p.(Trp353^a^)	stopgain	Shamanka II, Russia	3818
c.3817C>T	p.(Gln1273^a^)	stopgain	Czech Republic	NA
*BRCA2*				
c.9004G>A	p.(Glu3002Lys)	nonsynonymous SNV	Kyordyughen, Russia	4603
c.7975A>G	p.(Arg2659Gly)	nonsynonymous SNV	Budakalász-Luppa csárda, Hungary	5257
c.8488-1G>A	p.(=)	.	Uybat V, Russia	4329
c.9382C>T	p.(Arg3128^a^)	stopgain	Nesterivka, Ukrayina	8610
c.7007G>A	p.(Arg2336His)	nonsynonymous SNV	Shamanka II, Russia	6386

^a^before present

## Discussion

Portuguese played a unique role in modern human admixture through its multi-century’s global colonization across Africa, Asia and America. The global distribution of Portuguese genetics provides an attract model to study the impact of admixture on human health. *BRCA* PVs are well determined as the genetic predisposition for high cancer risk. They provide valuable genetic markers to trace the transmission of pathogenic variation between human populations by admixture. By tracing the distribution of *BRCA* PVs originated in Portuguese in the Portuguese heritage-enriched Brazilian population, we observed high sharing of Portuguese *BRCA* PVs in Brazilian population, as represented by the Portuguese founder PV of *BRCA1* c.2037delinsCC, *BRCA1* c.3331_3334del, and *BRCA2* c.156_157insAlu.

There are three models in explaining the genetic predisposition contributing to disease risk. Model 1: common variants contributing to common disease risk; model 2: low-frequent variants contributing to intermediate disease risk; and model 3: rare-frequent variants contributing to high disease risk [62]. For the *BRCA* PVs shared between Portuguese and Brazilian populations, they were either no or at extremely low frequencies of 0.00001 as reported in GnomAD (Fig. 3A). Therefore, these shared *BRCA* PVs are all rare variants, and the Model 3 fits the best to explain the cancer risk caused by these shared PVs. Genetic factor contributes 5–10% of breast cancer risk. Around 50% of the genetic factor is currently known that about 30% is by the rare *BRCA* PVs [63], 10-15% by the low frequent PVs in other genes such as *PTEN, TP53*, *CHEK2, PALB2* and *STK11* [64], and the 1-5% by the common variants. The rare and low frequent variants are often identified in cancer patients, the common variants are often identified in other genes by GWAS analysis at the population level. The rare variants in *BRCA* and low frequency variants in other genes often have higher penetrant in causing high cancer risk and high values for clinical applications, whereas the common variants often have low penetrant in causing low disease risk, which are often used for theoretical explanation of disease risk but not used for clinical applications. Nearly all *BRCA* PV-caused breast cancer is monogenic, implying that *BRCA* PV alone is sufficient to cause high cancer risk. The rarity of the *BRCA* PVs has an important clinical implication that individual-based approaches need to be taken to identify the PV carriers.

The three Portuguese-*BRCA* founder PVs of *BRCA1* c.2037delinsCC, *BRCA1* c.3331_3334del, and *BRCA2* c.156_157insAlu contribute nearly 60% of Portuguese *BRCA* PV carriers (Table 1B). The situation is similar to the three founder PVs of *BRCA1* c.68_69delAG, c.5266dupC, and *BRCA2* c.5946delT in 1 of 40 Ashkenazi Jews [65], and the three *BRCA1* founder PVs of C61G, 4153delA and 5382insC for 80% of the pathogenic *BRCA1* PV carriers in Polish population [66]. Although *BRCA1* c.3331_3334del is a founder variants in Portuguese, it is also present in the Iberia population [67]. Therefore, attention needs to pay in interpreting *BRCA1* c.3331_3334del as Portuguese-specific. *BRCA2* c.156_157insAlu accounted 46% of *BRCA* PVs in the Portuguese cancer cohort in our current study, much higher than the originally reported one-fourth, 33.6% or 37.9% [68–70]. This is likely due to the larger size of the combined patients than those in the individual original studies. Further, *BRCA2* c.156_157insAlu cannot be detected by regular PCR-sequencing approach but requires the use of two independent PCR with specific primers for sequencing. This feature suggests that the actual prevalence of *BRCA2* c.156_157insAlu could be higher than currently known. The presence of the three Portuguese founder variants in Brazilian provides strong evidence for their Portuguese origin. For other shared variants, some could occur coincidently in Portuguese and Brazilian, although the possibility may not be high considering their highly Portuguese-specific nature.

Ethnic specificity of PVs in cancer predisposition genes reflects genetic diversity and environmental adaptation of different ethnic populations, and can contribute to different susceptibility to disease risk in different populations [32, 71–73]. Ethnic specificity is contributed by the factors of evolution selection, bottleneck effects, genetic drift, and founder variation etc. during human evolution. Specifically, human *BRCA* PVs mostly arose in recent thousand years before present [74], and modern admixture initiated mostly in only a few hundreds of years ago. Referring to the *BRCA* PVs in a donor ethnic population should facilitate the identification of the PV carriers in the recipient population.

In conclusion, our study provides evidence that admixture can indeed impact on human health, in this case the cancer susceptibility, through transferring pathogenic variation from donor population to recipient population. While our study tested only *BRCA* PVs, It would be interesting to know if similar situation could be present in other disease risk genes.

## Materials and methods

### Sources and annotation of *BRCA* variation data in Portuguese and Brazilian populations

We performed a comprehensive literature search in PubMed and Google Scholar by using the terms “*BRCA1*”, “*BRCA2*”, “Portugal”, “Portuguese”, “Brazil”, “Brazilian” “variant”, and “mutation” in English and Portuguese, to identify the sources reporting *BRCA* variants originated from Portuguese and Brazilian population. We extracted the *BRCA* variation information from the original sources, including cDNA variant designation, amino acid designation, variant type, tested sample size, carrier number and cancer types. The collected *BRCA* variants were standardized following the human genome variation society (HGVS) nomenclature using the following references sequences: human genome reference: GRCh38.14(GCF_000001405.40); *BRCA1*: HGVSg: NC_000017.11, HGVSc: NM_007294.4, HGVSp: NP_009225.1; *BRCA2*: HGVSg: NC_000013.11, HGVSc: NM_000059.4, HGVSp: NP_000050.2. Variants were annotated by using ANNOVAR. Clinical class of the variants was based on ClinVar classification [75]. The carrier numbers for the same variants of different studies were combined to calculate carrier frequency in the tested cases. "1" was assigned for the variants without carrier information in the original sources. GnomAD v.3.1.2 database was used to search for variation frequency and Portuguese-specific *BRCA* PVs [76].

### Database construction

Two open-access databases, dbBRCA-Portuguese and dbBRCA-Brazilian, were constructed to host the *BRCA* variant data from Portuguese and Brazilian populations following the procedures [18]. Briefly, the databases were designed using the Linux operating system (CentOS 7, (https://www.centos.org/)). User searching and retrieval requests were performed by MySQL relational database management (5.6.50, (https://www.mysql.com/)) and PHP (version 7.3, (https://www.php.net/)). Web front-end languages (HTML, CSS, and JavaScript) were used to implement a user interface for the databases.

#### Ancient human genomic data analysis

The process followed the procedures [77]. Briefly, ancient human genome sequences were downloaded from the Allen Ancient DNA Resource including 5,047 ancient humans dated between 45,045 and 100 years before present (BP) (version 50.0, https://reich.hms.harvard.edu/allen-ancient-dna-resource-aadr-downloadable-genotypes-present-day-and-ancient-dna-data, accessed October 19, 2021). *BRCA* variants in ancient humans were called by SAMtool and annotated by ANNOVAR, and compared with the 78 *BRCA* PVs shared between Portuguese and Brazilian. The location and dated age of ancient human individuals carrying the matched *BRCA* PVs were extracted from the original data.

### Supplementary Information

**Additional file 1: Table S1.** Global human population with Portuguese heritage.

**Additional file 2: Table S2.** List of Portuguese *BRCA* variants.

**Additional file 3: Table S3.** Variation types of Portuguese *BRCA* variants.

**Additional file 4: Table S4.** Large structural changes in Portuguese *BRCA* variants.

**Additional file 5: Table S5.** Portuguese-specific *BRCA* PVs.

**Additional file 6: Table S6.** List of Brazilian *BRCA* PVs.

## Data Availability

The *BRCA* variation data generated from this study are available at: https://genemutation.fhs.um.edu.mo/dbbrca-portuguese/, and https://genemutation.fhs.um.edu.mo/dbbrca-brazilian.
